# Supplementary material for: Discovery of Novel Non-Oxime Reactivators Showing In Vivo Antidotal Efficiency for Sarin Poisoned Mice
Source: Molecules. 2022 Feb 7;27(3):1096. doi: 10.3390/molecules27031096 (PMC8840479; doi:10.3390/molecules27031096)
Supplement: Supplementary file 1 [file molecules-27-01096-s001.zip › molecules-1564893-supplementary.pdf]

Supplementary Materials

Discovery of novel non-oxime reactivators showing *in vivo* antidotal efficiency for sarin poisoned mice

Zhao Wei [1,*], Xinlei Zhang [1], Huifang Nie [1], Lin Yao [1], Yanqin Liu [2], Zhibing Zheng [2,*], and Qin Ouyang [3,*]

[1] Department of Medicinal Chemistry, School of Pharmacy, Air Force Medical University, Xi'an 300071, China; yxxxxjys@fmmu.edu.cn (X.Z.); 13572002997@163.com (H.N.); yaolinpharf@126.com (L.Y.)

[2] Institute of Pharmacology and Toxicology, Academy of Military Medical Sciences, Beijing 100850, China; lyqf819@aliyun.com (Y.L.); zzbcaptain@aliyun.com (Z.Z.)

[3] Department of Medicinal Chemistry, School of Pharmacy, Third Military Medical University, Chongqing 400038, China

* Correspondence: weizhaobruce@163.com (Z.W.); zzbcaptain@aliyun.com (Z.Z.); ouyangq@tmmu.edu.cn (Q.O.)

1. NMR and HR-MS Spectra for R4, L6R1, L10R1, L6R4 and L10R4

3-((1H-imidazol-1-yl)methyl)-4-hydroxybenzaldehyde (R4) ^{1}H NMR (DMSO, 400 MHz)

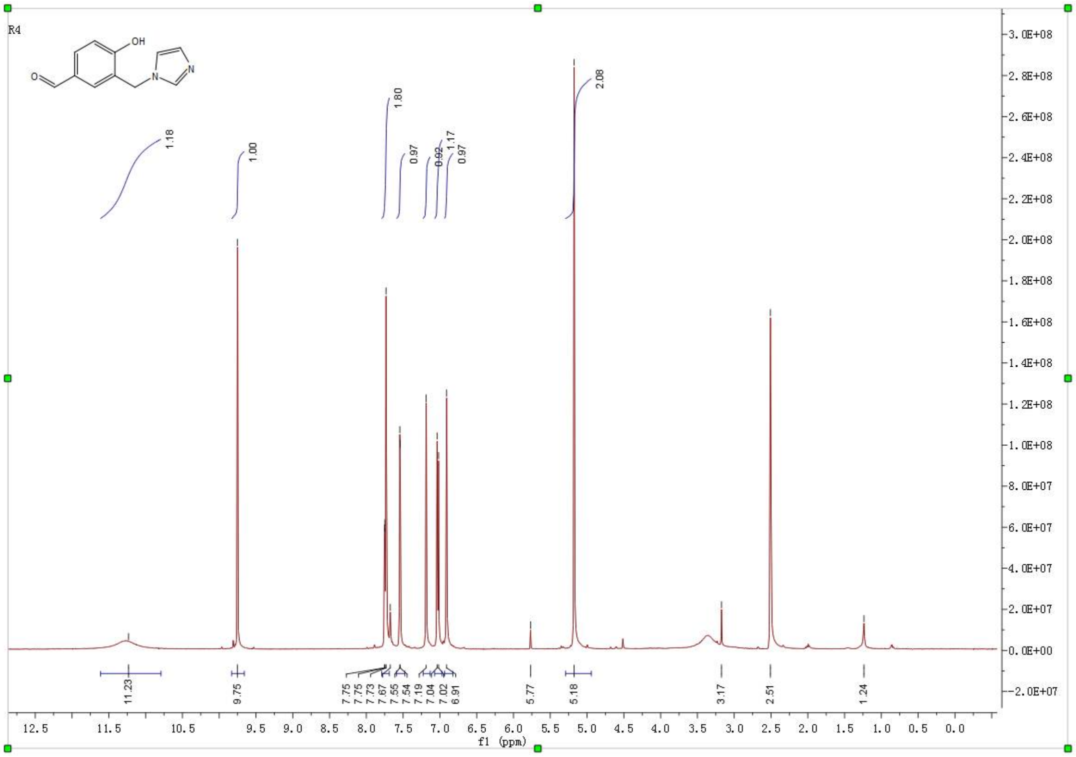

4-((3-((diethylamino)methyl)-4-hydroxybenzyl)amino)benzamide (L6R1)

^{1}H NMR (DMSO, 400 MHz) and HRMS (ESI^{+})

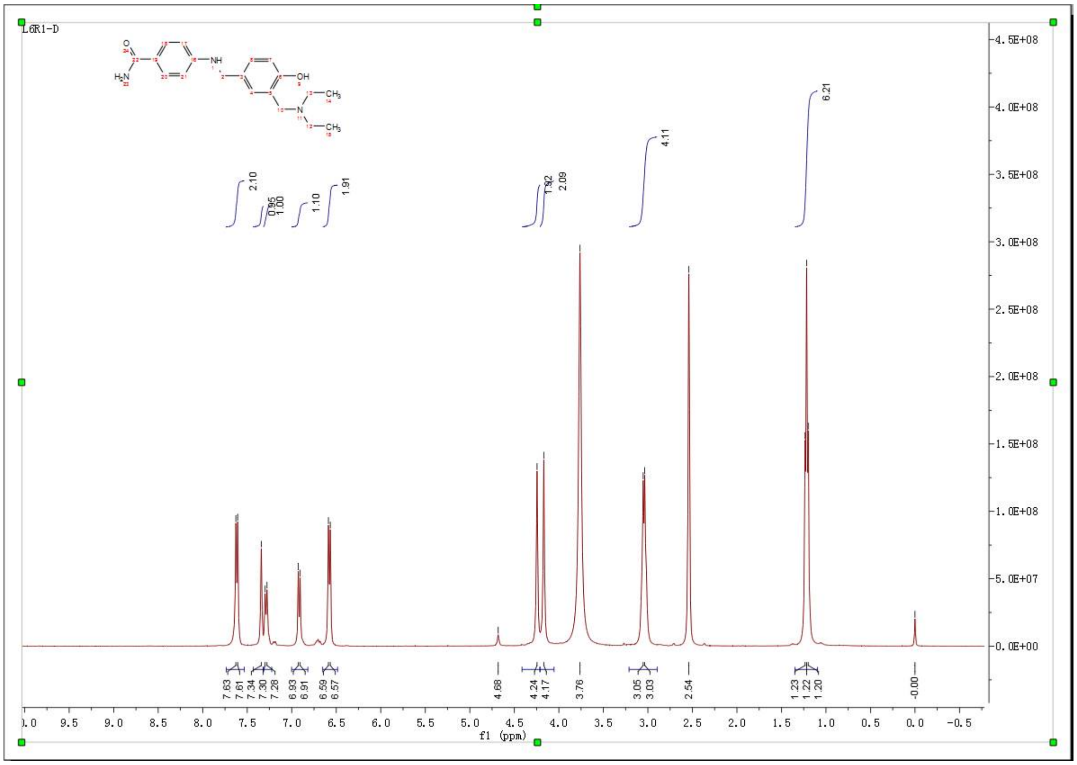

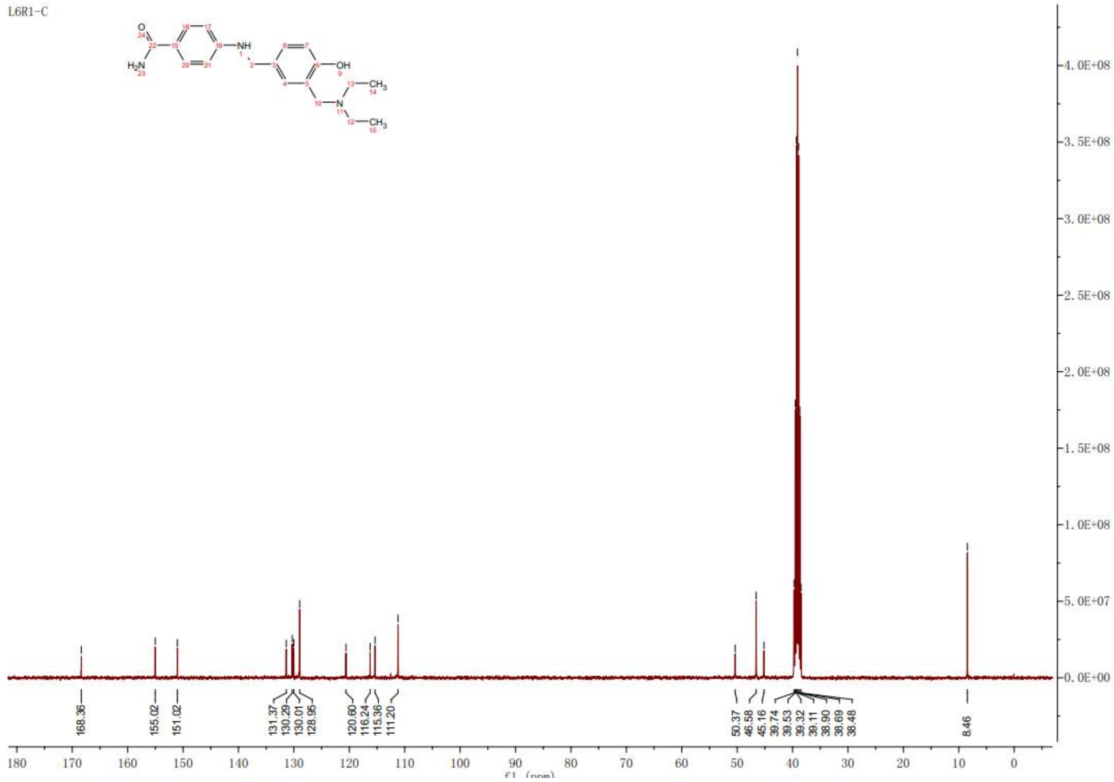

Qualitative Analysis Report

**Data Filename**	5553.d	**Sample Name**	L6R1
**Instrument Name**	TOF G6230A	**Acquired Time**	2021-09-28
**Acq Method**	YCL.M	**Acquired SW**	6200 series TOF/6500 series
**IRM Calibration Status**	**Success**		
**User Chormatograms**			

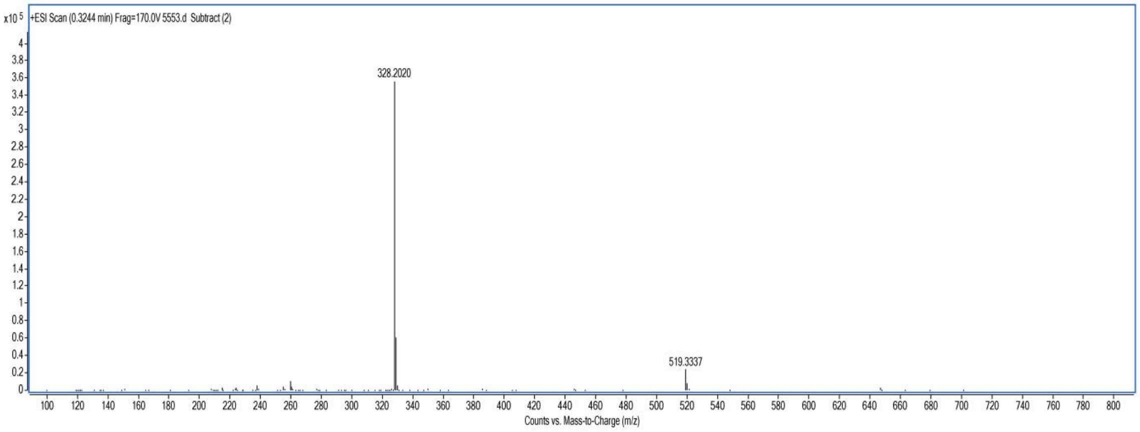

4-(((1H-pyrazolo[3,4-b]pyridin-3-yl)amino)methyl)-2-((diethylamino)methyl)phenol (L10R1):

^{1}H NMR (DMSO, 400 MHz) and HRMS (ESI^{+})

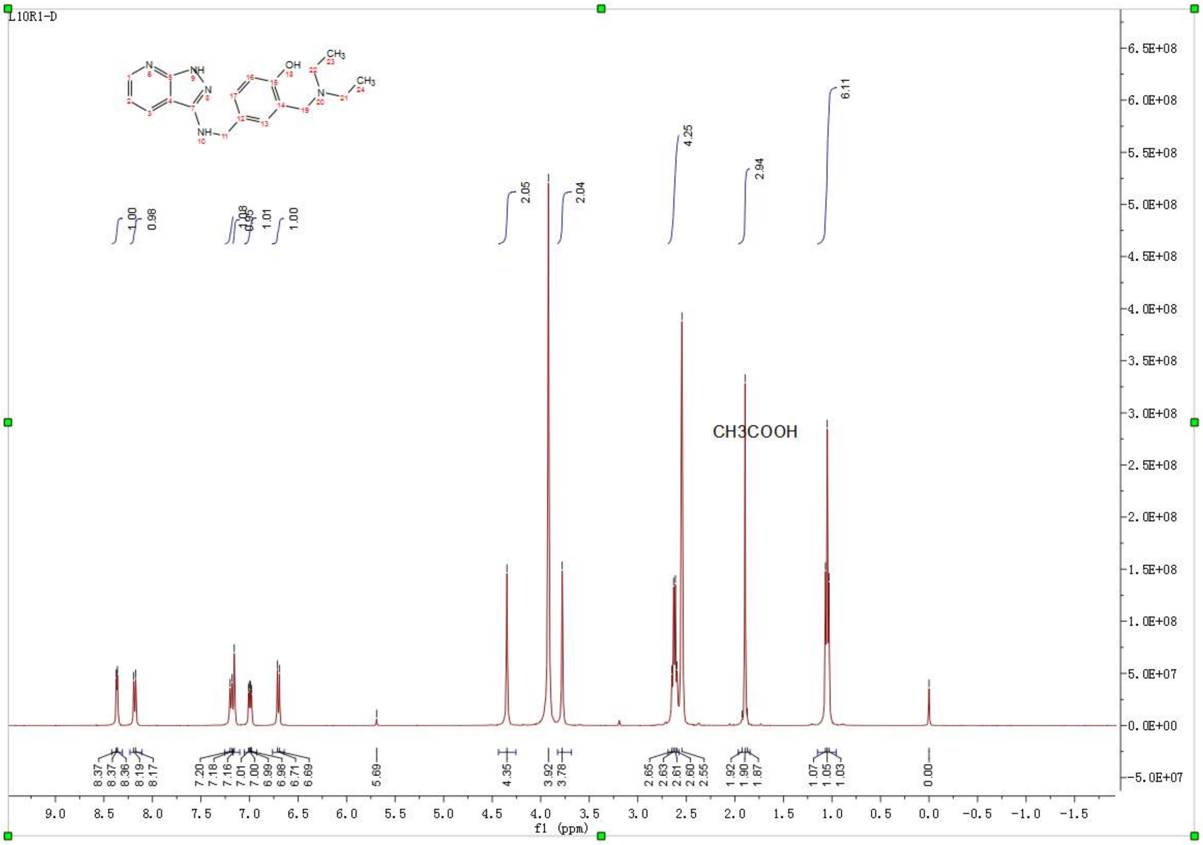

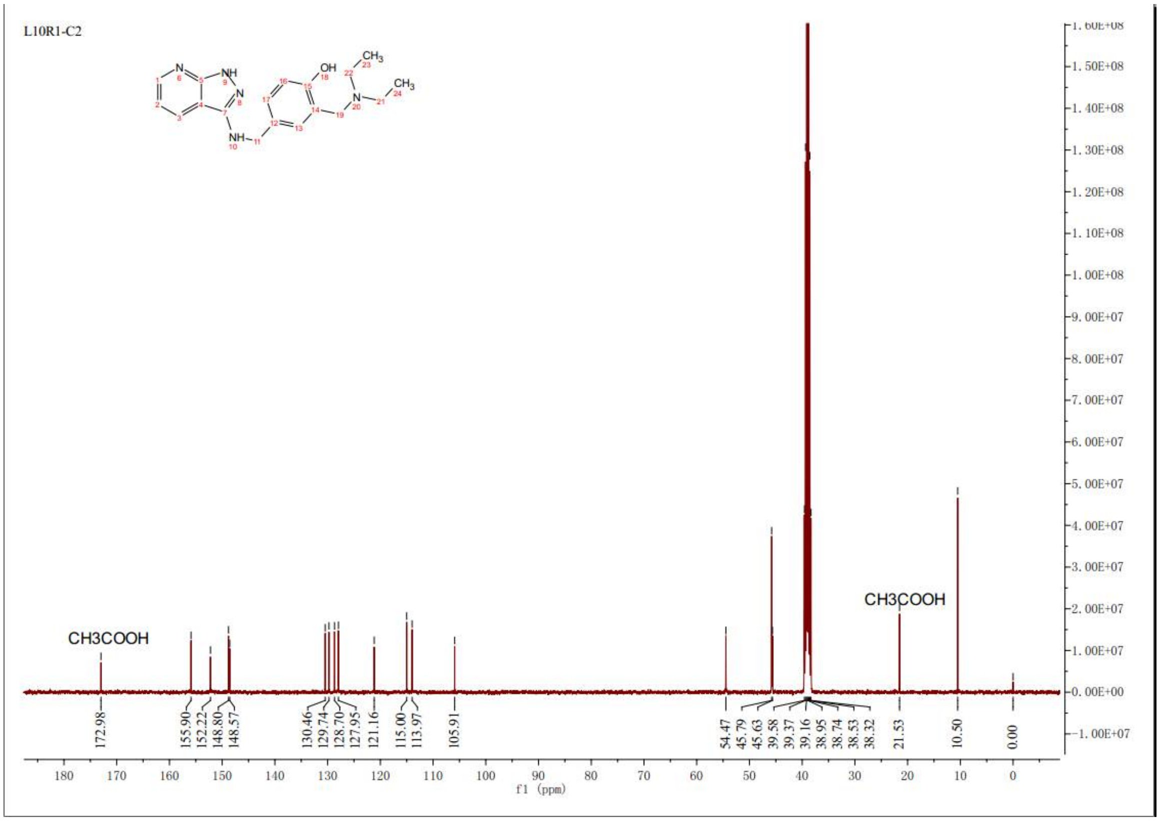

Qualitative Analysis Report

**Data Filename**	5552.d	**Sample Name**	L10R1-2
**Instrument Name**	TOF G6230A	**Acquired Time**	2021-09-28
**Acq Method**	YCL.M	**Acquired SW**	6200 series TOF/6500 series
**IRM Calibration Status**	**Success**		
**User Chormatograms**			

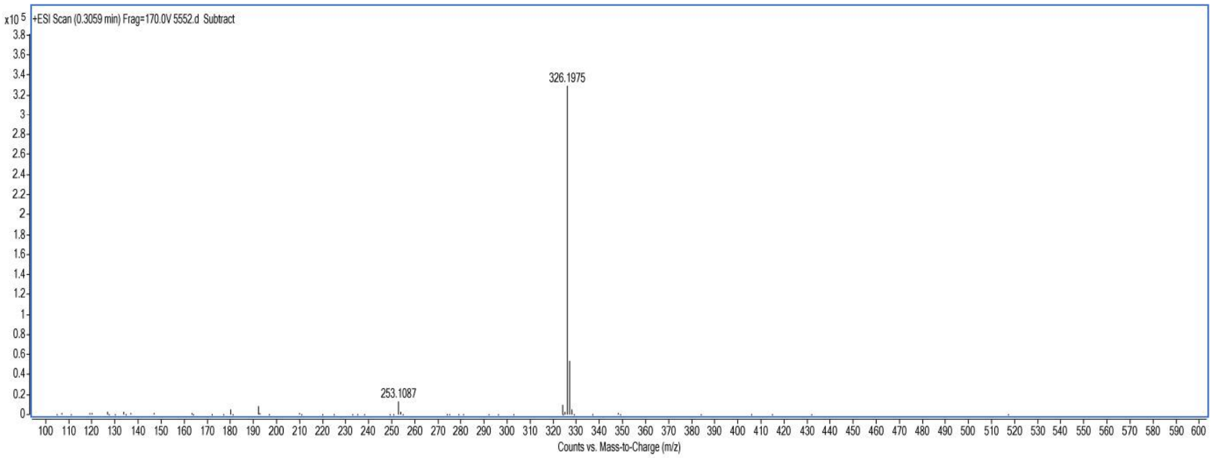

4-((3-((1H-imidazol-1-yl)methyl)-4-hydroxybenzyl)amino)benzamide (L6R4):

^{1}H NMR (DMSO-D$_2$O, 400 MHz) and HRMS (ESI^{+})

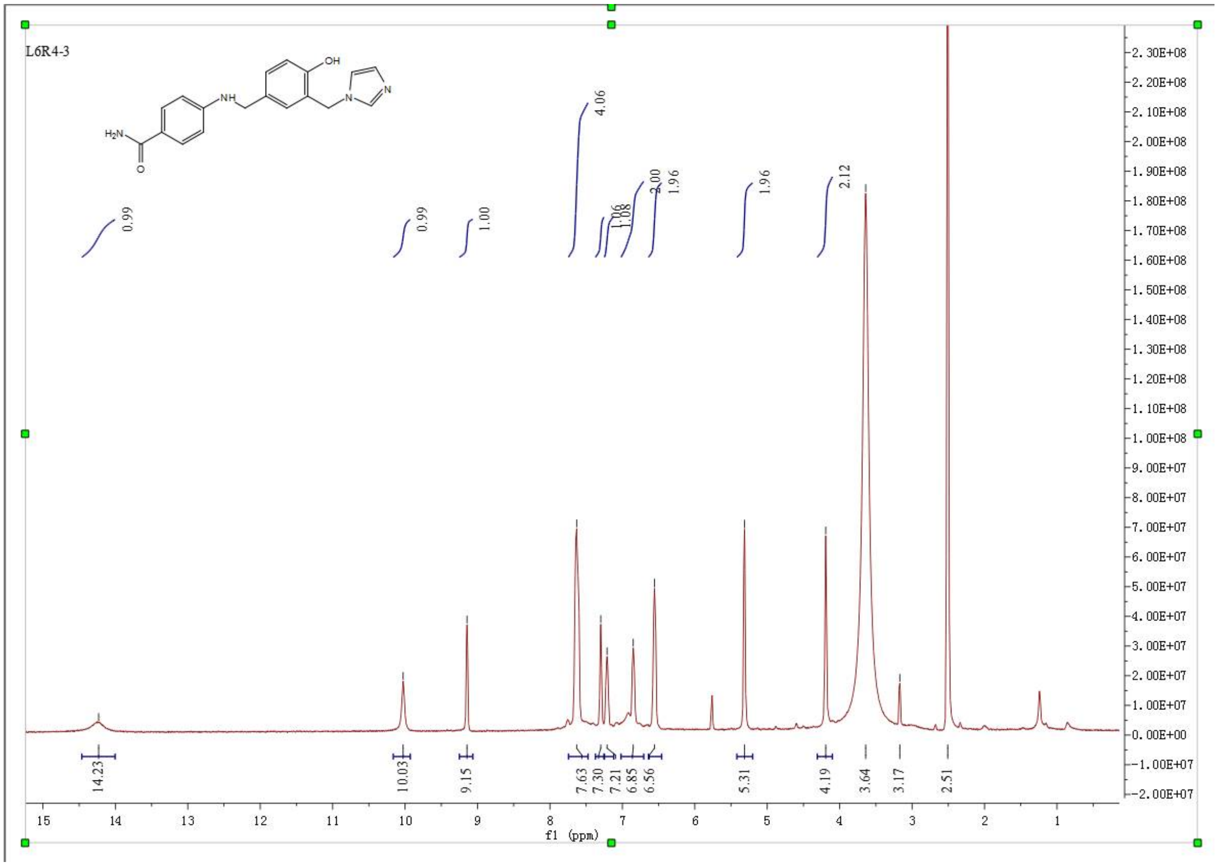

Qualitative Analysis Report

**Data Filename**	5526.d	**Sample Name**	L6R4
**Instrument Name**	TOF G6230A	**Acquired Time**	2021-09-27
**Acq Method**	YCL.M	**Acquired SW**	6200 series TOF/6500 series
**IRM Calibration Status**	Success		
**User Chormatograms**			

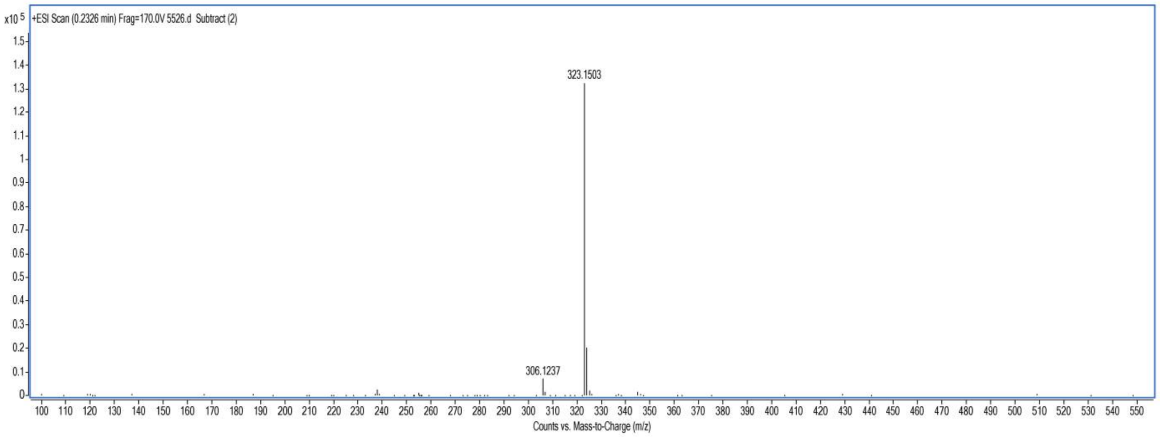

2-((1H-imidazol-1-yl)methyl)-4-(((1H-pyrazolo[3,4-b]pyridin-3-yl)amino)methyl)phenol (L10R4):

^{1}H NMR (DMSO-D_2O, 400 MHz) and HRMS (ESI^{+})

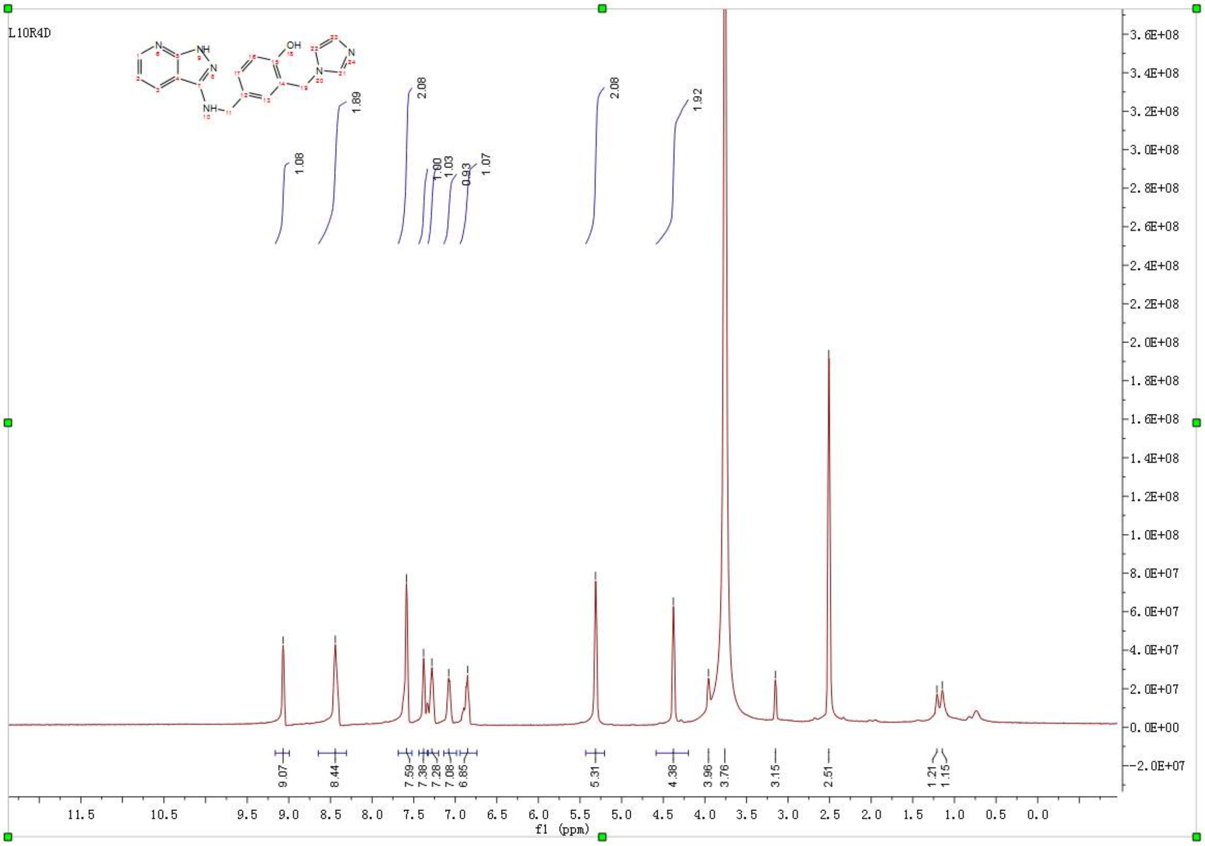

Qualitative Analysis Report

**Data Filename**	5525.d	**Sample Name**	L10R4
**Instrument Name**	TOF G6230A	**Acquired Time**	2021-09-27
**Acq Method**	YCL.M	**Acquired SW**	6200 series TOF/6500 series
**IRM Calibration Status**	**Success**		
**User Chormatograms**			

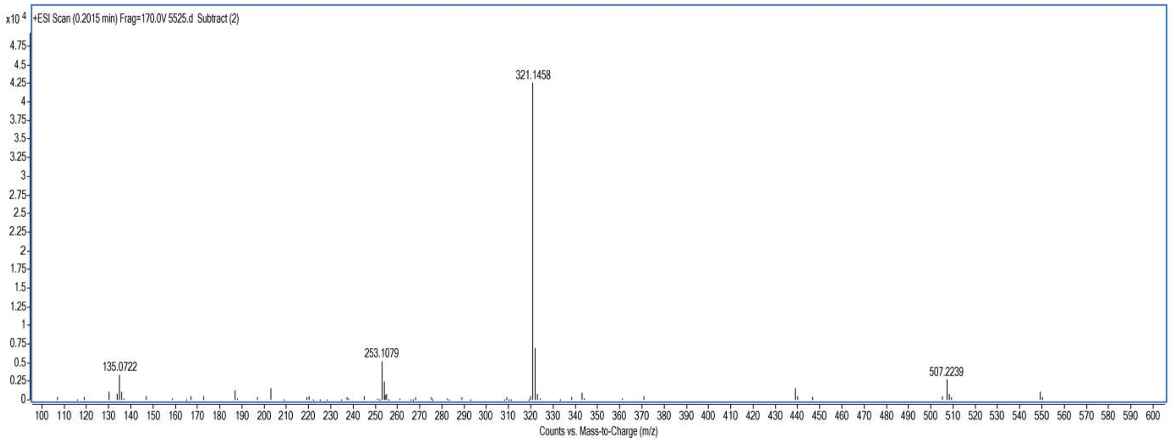

2. Biological evaluation

Table S1. %Reactivation of non-oxime reactivators for VX and sarin inhibited hAChE

VX-hAChE				
	**100 µM**	**50 µM**	**25 µM**	**10 µM**
**HI-6**	74.02±2.28	64.61±0.72	49.38±1.36	32.72±1.05
**L6R1**	68.40±0.80	48.26±1.36	29.74±1.82	13.21±1.07
**L10R1**	-	-	20.29±1.54	20.76±0.10
**L6R4**	90.62±2.47	68.63±3.39	34.55±0.25	19.12±2.12
**L10R4**	96.42±0.90	92.85±4.21	75.66±2.13	44.74±1.10
sarin-hAChE				
	**100 µM**	**50 µM**	**25 µM**	**10 µM**
**HI-6**	51.20±0.69	46.27±0.88	28.47±1.00	21.25±6.86
**L6R1**	22.72±0.88	13.90±0.18	5.86±3.25	5.83±0.86
**L10R1**	-	9.12±0.70	7.51±0.24	7.91±2.58
**L6R4**	30.38±1.58	21.84±1.39	14.99±0.40	13.68±0.78
**L10R4**	22.52±0.24	17.10±0.09	7.94±1.94	6.33±0.21

Table S2. Selected observed first-order rate constant K_{obs} of HI-6 and novel non-oxime reactivators.

	**VX-hAChE/k_{obs} (10^{-3}min^{-1})**			**Sarin-hAChE/k_{obs}(10^{-4}min^{-1})**			**IC$_{50}$(µM)**
	**50 µM**	**25 µM**	**10 µM**	**100 µM**	**50 µM**	**20 µM**	
**HI-6**	20.8±1.4	12.3±0.7	6.7±0.3	137.4±8.4	121.0±6.4	65.3±3.0	668±61
**L6R1**	11.8±1.1	5.8±0.6	2.4±0.2	41.2±2.3	22.8±1.4	10.7±0.9	439±18
**L10R1**	-	4.0±0.2	4.3±0.2	-	14.2±0.8	13.8±1.1	30.9±0.4
**L6R4**	191.1±21.9	56.8±4.0	13.6±0.9	68.4±4.0	41.3±3.6	22.7±1.3	402±19
**L10R4**	89.7±10.4	31.4±2.7	10.6±0.6	47.2±2.0	31.1±1.6	15.8±1.0	483±33

Experiments were performed in duplicate at 25 °C in phosphate buffer (0.10 M, pH 7.4), data shows the nonlinear fitting results and

standard deviation.

Plot of k_{obs} *vs* concentrations of **HI-6** and new synthesized compounds.

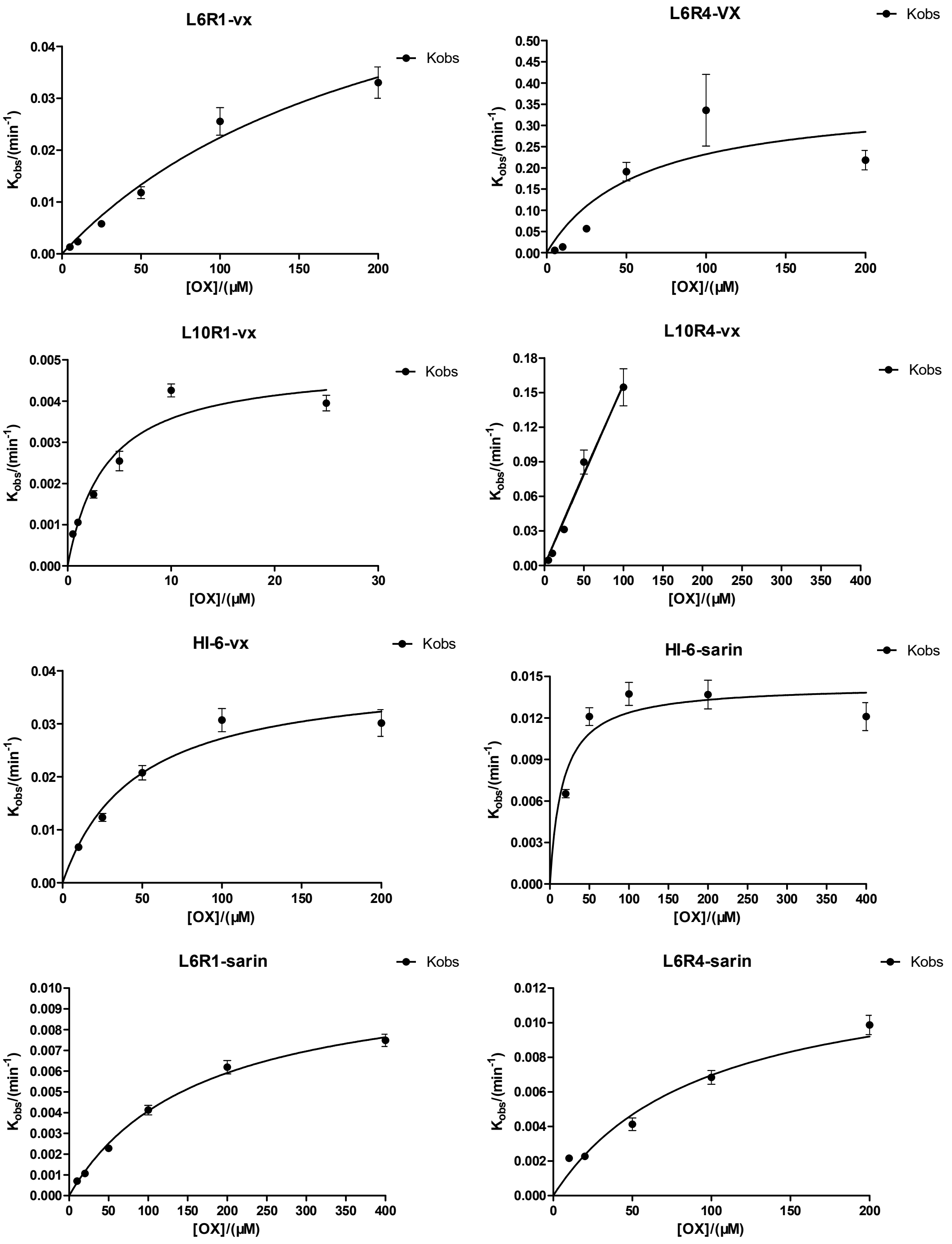

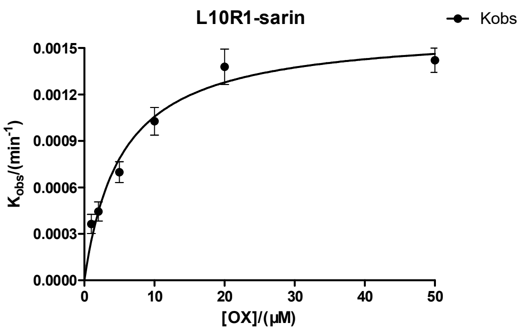
L10R1-sarin
Kobs
0.0015
0.0012
0.0009
0.0006
0.0003
0.0000
$K_{obs}/(min^{-1})$
0
10
20
30
40
50
[OX]/(μM)

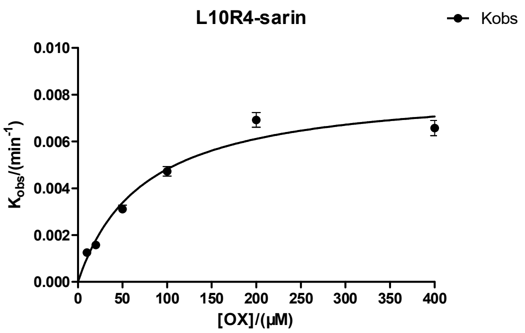
L10R4-sarin
Kobs
0.010
0.008
0.006
0.004
0.002
0.000
$K_{obs}/(min^{-1})$
0
50
100
150
200
250
300
350
400
[OX]/(μM)
